# Supplementary material for: Nasal Polyposis Quality of Life (NPQ): Development and Validation of the First Specific Quality of Life Questionnaire for Chronic Rhinosinusitis with Nasal Polyps
Source: Healthcare (Basel). 2022 Jan 28;10(2):253. doi: 10.3390/healthcare10020253 (PMC8871881; doi:10.3390/healthcare10020253)
Supplement: Supplementary file 1 [file healthcare-10-00253-s001.zip › healthcare-1533501-supplementary.pdf]

healthcare

Table S1. Item generation: results of items generation phase

1. Pain
2. Being nervous
3. Having to do clinical examinations
4. Having disturbed sleep
5. Swelling
6. Being worried about my health
7. Feeling embarassed due to my physical appearance
8. Being limited in my life activities
9. Lack of symptoms control
10. Being bothered to take medications
11. Being weary
12. Sleep problems
13. Being upset for my health condition
14. Feeling uncomfortable in social relationship
15. Smell disorders
16. Having to spend money
17. Dry mouth
18. Restricted in sport activities
19. Bad breath
20. Restricted in physical activities of daily life
21. Wake up during night to drink
22. Having a bad taste in the mouth
23. Difficulty enjoyng food and wine
24. Feeling irritable
25. Difficulty concentrating
26. Feeling tired
27. Loss of smell
28. Anxiety
29. Feeling uncomfortable with other people
30. Feeling embarassed due to the symptoms
31. Kneaded mouth
32. Being worried
33. Feeling embarassed in social life
34. Dark circles
35. Swollen face
36. Having to do CT scans
37. Hearing problems
38. Being bothered by medication side effects
39. Being bothered for the possibility of surgery
40. Being annoyed by frequent medical control
41. Feeling stressed

42. Feeling to have poor disease control
43. Nasal voice
44. Snoring
45. Having to do invasive clinical examinations
46. Having difficulties in intimate life
47. Being worried by long term drug efficacy
48. Kissing difficulty
49. Having difficulties in controlling symptoms
50. Fear that the problem will recur
51. Afraid not to notice to stink (when you sweat)
52. Facial pain
53. Headache
54. Make less than you would like

The validated Italian version of the questionnaire

Il presente questionario ha lo scopo di valutare l'impatto della rinosinusite cronica con poliposi nasale sulla vita quotidiana.

Indichi con *una* crocetta quanto è stato disturbato dalla presenza di ognuno dei seguenti problemi nel corso delle ultime 2 settimane a causa della rinosinusite cronica con poliposi nasale

1) Disturbi del sonno

Per niente Un po' Abbastanza Molto Moltissimo

2) Avere la gola asciutta

Per niente Un po' Abbastanza Molto Moltissimo

3) Essere limitati nello svolgere attività sportive

Per niente Un po' Abbastanza Molto Moltissimo

4) Avere un alito sgradevole

Per niente Un po' Abbastanza Molto Moltissimo

5) Fare fatica a gustare il cibo e il vino

Per niente Un po' Abbastanza Molto Moltissimo

6) Sentirsi irritabile

Per niente Un po' Abbastanza Molto Moltissimo

7) Essere preoccupati per gli effetti collaterali dei farmaci

Per niente Un po' Abbastanza Molto Moltissimo

8) Sentirsi in imbarazzo in situazioni sociali

Per niente Un po' Abbastanza Molto Moltissimo

9) Avere una voce nasale

Per niente Un po' Abbastanza Molto Moltissimo

10) Essere preoccupato per questa patologia

Per niente Un po' Abbastanza Molto Moltissimo

11) Sentire di avere poco controllo sulla malattia

Per niente Un po' Abbastanza Molto Moltissimo

12) Temere di non accorgersi di avere un odore sgradevole addosso (ad esempio quando si suda)

Per niente Un po' Abbastanza Molto Moltissimo

13) Avere mal di testa

Per niente Un po' Abbastanza Molto Moltissimo

14) Temere che il problema si ripresenterà

Per niente Un po' Abbastanza Molto Moltissimo

15) Essere preoccupato per la possibilità di un intervento chirurgico

Per niente Un po' Abbastanza Molto Moltissimo

16) Sentirsi stressati

Per niente Un po' Abbastanza Molto Moltissimo

17) Russare di notte

Per niente Un po' Abbastanza Molto Moltissimo

18) Avere difficoltà a concentrarsi

Per niente Un po' Abbastanza Molto Moltissimo

19) Fare fatica a sentire gli odori

Per niente Un po' Abbastanza Molto Moltissimo

20) Sentirsi in imbarazzo per i sintomi

Per niente Un po’ Abbastanza Molto Moltissimo

21) Avere un cattivo sapore in bocca

Per niente Un po’ Abbastanza Molto Moltissimo

22) Sentirsi la bocca impastata

Per niente Un po’ Abbastanza Molto Moltissimo

23) Sentirsi stanco

Per niente Un po’ Abbastanza Molto Moltissimo

24) Essere preoccupato che i farmaci a lungo andare siano meno efficaci

Per niente Un po’ Abbastanza Molto Moltissimo

25) Sentirsi a disagio con le altre persone

Per niente Un po’ Abbastanza Molto Moltissimo

26) Fare fatica a controllare i sintomi

Per niente Un po’ Abbastanza Molto Moltissimo

27) Rendere meno di quanto vorrebbe

Per niente Un po’ Abbastanza Molto Moltissimo
